# Analysis of dose–TSH response effect of levothyroxine soft-gel formulation

**DOI:** 10.3389/fendo.2024.1340204

**Published:** 2024-02-21

**Authors:** Pierpaolo Trimboli, Tommaso Piticchio, Zeno Dadda, Ilaria Stramazzo, Elena Gamarra, Lorenzo Ruinelli, Camilla Virili

**Affiliations:** ^1^ Servizio di Endocrinologia e Diabetologia, Ospedale Regionale di Lugano, Ente Ospedaliero Cantonale, Lugano, Switzerland; ^2^ Facoltà di Scienze Biomediche, Università della Svizzera Italiana, Lugano, Switzerland; ^3^ Endocrinology Section, Department of Clinical and Experimental Medicine, Garibaldi Nesima Hospital, University of Catania, Catania, Italy; ^4^ Endocrinology Section, Department of Medico-Surgical Sciences and Biotechnologies, Sapienza University of Rome, Latina, Italy; ^5^ Team Data Science and Research, Area ICT, Ente Ospedaliero Cantonale, Bellinzona, Switzerland; ^6^ Clinical Trial Unit, Ente Ospedaliero Cantonale, Bellinzona, Switzerland

**Keywords:** thyroid, hypothyroidism, soft-gel, CAPS, TSH, levothyroxine, dose

## Abstract

**Background:**

Hypothyroidism is treated with daily levothyroxine (LT4). In recent years, soft gel caps of LT4 (LT4-C) have been commercialized, and their performance has been optimized. Since guidelines recommend dose LT4 according to the tablet preparation efficacy, the present study was undertaken to obtain data about the daily requirement, normalized per body weight, of LT4-C.

**Methods:**

Patients undergoing LT4-C after total thyroidectomy and radioiodine treatment for differentiated thyroid carcinoma were selected. There was no specific indication of suppression of TSH (i.e., <0.5 or <0.1 mIU/L). Patients were required to maintain a stable LT4 dose during the study period. Patients with interfering factors were excluded from this study.

**Results:**

Thirty patients were enrolled (18 females and 12 males; median age, 50 years; median body weight, 71 kg; median LT4-C dose, 1.71 µg/kg/day). The analysis of patient age did not reveal any differences. The LT4-C dose correlated with free-T4 p = 0.03), but not with TSH (p = 0.42) and free-T3 (p = 0.13). TSH was <1.0 mIU/L in 90% of the cases. The LT4-C dose–TSH response effect was analysed by probit regression model: the probability to achieve TSH <1.0 mIU/l was 99% with a dose of 1.84 (95%CI 1.57–2.12) µg/kg/day, 75% with a dose of 1.38 µg/kg/day (95%CI 1.17–1.59), and 50% with a dose of 1.20 (95%CI 0.96–1.43). At ROC curve analysis, the most accurate cut-off of LT4-C dose to achieve TSH <1.0 mIU/l was 1.53 ug/kg/day with 70% sensitivity and 100% specificity.

**Conclusions:**

Athyreotic patients can be initially treated with an LT4-C dose lower than previously stated. Therefore, further prospective studies are warranted.

## Introduction

Hypothyroidism is a frequent non-communicable condition that represents the second most common endocrine disorder after diabetes mellitus (1). The treatment of choice for hypothyroid patients is represented by the daily consumption of sodium levothyroxine (LT4); its tablet preparation (LT4-T), first commercialized in the 60s, is still the most used pharmaceutical form (2, 3). Currently, two other preparations of LT4, the liquid solution and soft gel capsules (LT4-C), are available in the market in several countries. Despite different formulations sharing the same active ingredient, relevant differences exist among them (4). While tablet preparation requires a disintegration/dissolution process to release the active ingredient in the stomach, the liquid solution does not require specific steps, and capsules are rapidly melted in the gastric lumen (5–7). This means that while the performance of LT4-T closely depends on the characteristic of the gastric environment, the pharmacokinetics of liquid and cap preparations are less likely to be impaired by gastric status (8, 9). Several studies demonstrated that LT4-T efficacy can be reduced by food intake, medications and gastric disorders influencing gastric pH, thus decreasing LT4 bioavailability (i.e., *Helicobacter pylori* gastritis, atrophic gastritis, proton-pump inhibitors, aluminium-containing antacids, calcium carbonate, sucralfate, etc.) (10, 11). On the other hand, several studies have shown that patients with suboptimal results on LT4-T can clinically and biochemically benefit once switched to liquid or cap preparations at unchanged or lower daily doses (12–17). Consequently, clinicians have recently moved their preferences in favor of non-tablet preparations (18–20).

Since LT4-T was the first to be commercialized and there is more experience in its use, international guidelines recommend the use of this preparation, only occasionally mentioning the opportunity of treatment with novel formulations in selected subpopulations of patients. American Thyroid Association (ATA) guideline suggests that “*hypothyroid patients with minimal endogenous thyroid function require* tablet *LT4 doses of 1.6–1.8 mcg/kg of actual body weight, although some studies estimate higher doses of 2.0–2.1 mcg/kg for some patient groups*” (3). Furthermore, a seminal review article on this topic by Biondi and Wartofsky suggested that “*larger doses are required in athyreotic patients compared with patients with residual thyroid function (thyroidectomized patients vs autoimmune hypothyroidism and radioiodine ablated patients*” (21). However, the available guidelines on this topic were prepared before most of the studies on the performance of these new formulations were published, and this fact raises the question of whether these recommendations may also apply to non-tablet preparations. To answer this question, one should evaluate LT4-C data collected during clinical practice, in a context where non-tablet preparations are largely prescribed, and physicians are confident about their use. Since Switzerland is the country where non-tablet preparations were first developed and considering that LT4-C has been commercialized several years ago with a mild difference in cost with respect to the tablet, the context of our institution seems to be ideal to address the issue.

Based on the above issues, the present study was undertaken to obtain solid information about the daily requirement (normalized per body weight) of non-tablet preparations. Because liquid preparation has been commercialized in Switzerland very recently, the present study aimed to evaluate the performance of LT4-C. Case selection included athyreotic patients followed up for differentiated thyroid carcinoma (DTC) who were treated to reach the target of low-normal TSH.

## Methods

### Study conduction

The study was conducted according to the STrengthening the Reporting of OBservational studies in Epidemiology (STROBE) statement (22). The checklist is reported as **Supplementary Material**.

### Institutional setting

Ente Ospedaliero Cantonale (EOC) is the major public health institution of the Tessin canton and includes the regional hospitals of major cantonal towns. The EOC Clinic for Endocrinology was present in all the hospitals. Additionally, EOC represents the highest-volume institution for thyroid diseases and surgery. After total thyroidectomy, DTC patients usually begin LT4 treatment at a fixed initial dose of 1.5 mcg/kg/day–1.6 mcg/kg/day. Later, patients were placed on follow-up, thyroid tests were monitored, and the LT4 dose was eventually adjusted according to blood results, subjective clinical benefit, and case-specific TSH target, when indicated. Noticeably, since LT4-C was commercialized in Switzerland several years ago, it has been largely used as a first-line treatment option. TSH levels were then verified following six-to-eight weeks. Upon the hormonal check, LT4 dose is adjusted, if needed, to achieve the TSH target that in DTC usually is represented by levels at least below 1.0 mIU/L or suppressed levels, when indicated in very high-risk cases (23) When TSH target is achieved, LT4 dose is maintained, and follow-up starts.

### Case selection

#### Inclusion criteria

The study included patients seen during the period between 2020 and 2023.

As major inclusion criteria, patients were followed up for DTC, underwent radioiodine treatment according to their individual features, were assessed as low-risk according to the ATA, and were on LT4-C therapy without specific indication to have suppressed TSH (i.e., <0.5 or <0.1 mIU/L) (23). The selection of this specific setting of cases was aimed at recruiting patients without even minimal endogenous thyroid function after surgery and subsequent ablation of thyroid remnants. Furthermore, they are usually followed by specialists to maintain the TSH value in a strictly tailored range, regardless of whether it needs to be suppressed. As other inclusion criteria, patients were required to maintain a stable LT4 dose during the study period, defined as the difference in dose not exceeding one decimal per kg, once euthyroidism was achieved.

#### Exclusion criteria

The exclusion criteria were: 1) patients taking drugs affecting thyroid tests (i.e., bromocriptine, somatostatin, oral contraceptive, lithium, glucocorticoid, sulfonamide, iron or iodine supplements, etc.); 2) patients having comorbidities interfering with intestinal LT4 absorption (i.e., celiac disease, Crohn disease, intestinal inflammatory diseases, post-bariatric surgery, etc.); 3) patients featuring major and/or chronic disorders (i.e., severe non-communicable diseases, obesity, major depression, infections); 4) pregnant or lactating patients; 5) patients recently undergone radiological examination (10, 21, 24).

### Levothyroxine formulation

The present study included only patients on LT4-C commercialized by the IBSA Institut Biochimique SA (Tirosint^®^).

### Laboratory measurement

Thyroid test data were collected during routine clinical practice. Patients underwent blood tests during their visits. Thyroid tests were performed using an Elecsys^®^ e601 platform (Roche Diagnostics). In this assay, TSH sensitivity was 0.006 mIU/L, with reference range of 0.30 mIU/L and 4.20 mIU/L. Free-T3 and free-T4 reference ranges are 3.1 pmol/L –6.8 pmol/L and 12 pmol/L –22 pmol/L, respectively.

### Parameters and measures

Data on sex, age, and anthropometric features of the selected cases were collected. TSH, free-T3, and free-T4 were evaluated, and the data reported in the text were referred to the last hormonal check. The LT4 dose reported in the text represents the median dose administered during the study period.

### Statistical analysis

Continuous parameter data are expressed as median and interquartile range (IQR). Data were compared using nonparametric statistical tests for paired or unpaired data, when indicated. The LT4 dose–TSH response effect was analyzed using a probit regression model to estimate the dose required to achieve TSH <1.0 mIU/L and the dose was expressed as a value with a 95% confidence interval (95%CI). Receiver operating characteristic (ROC) curve analysis was performed to identify the maximum Youden index (J) representing the most accurate LT4 dose to achieve TSH <1.0 mIU/L. Correlations were analyzed using the Spearman’s test. Statistical analysis was performed using ^©^ 2023 MedCalc Software Ltd.

## Results

**Figure 1:**
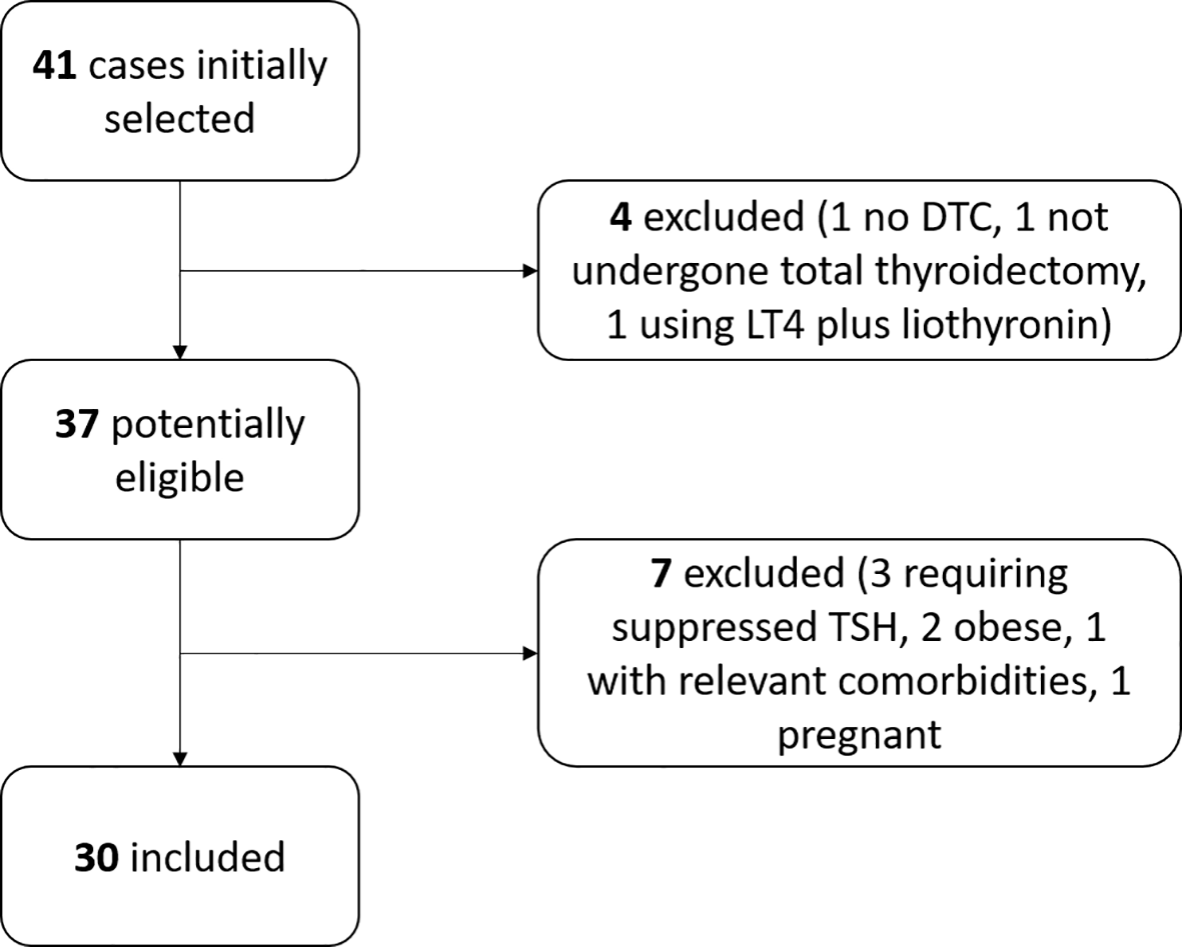
Flow diagram of case selection.

**Figure 2:**
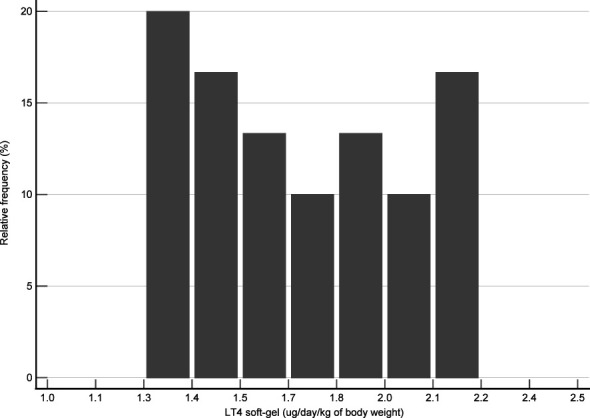
Distribution of cases according to their daily dose of LT4 per body weight. The LT4 dose represents the mean value assumed for each patient during the study period.

Forty-one DTC patients were initially identified in the institutional database. After using the inclusion and exclusion criteria, 30 patients were enrolled in the present study. **Figure 1** shows the flow diagram. This study included 18 females and 12 males. Anthropometric characteristics of the study groups are presented in **Table 1**. The distribution of patients according to the daily LT4 dose normalized by body weight is shown in **Figure 2**.

**Table 1:** General characteristics of the study group.

	Median	IQR
**Age, years**	50	43.70–60.00
**Body weight, kg**	71	62.00–82.50
**TSH, mIU/L**	0.13	0.04–0.62
**Free-T3, pmol/L**	4.60	4.10–5.22
**Free-T4, pmol/L**	23.7	20.25–26.20
**LT4 dose, µg/kg/day**	1.71	1.48–2.08
**Period of observation, months**	8.20	4.68–17.03

IQR, interquartile ranges.

The analysis of patients’ age did not reveal differences in the above anthropometric characteristics or hormonal thyroid data, although a slight decrease in daily LT4 requirement might be observed in older patients (>60 years), according to the reduction in hormonal clearance and lean body mass described in this life period (3, 22) (**Table 2**).

**Table 2:** Comparison between patients older or younger than 60 years.

	<60-y-old		>60-y-old		
	Median	IQR	Median	IQR	p
**Body weight, kg**	69	54.5–82.3	73	66.2–89.7	0.325
**TSH, mIU/L**	0.185	0.048–0.668	0.093	0.022–0.332	0.534
**Free-T3, pmol/L**	4.50	4.10–4.97	4.60	3.82–5.55	0.862
**Free-T4, pmol/L**	23.85	20.08–26.9	22.45	20.68–25.20	0.721
**LT4 dose, µg/kg/day**	1.785	1.51–2.08	1.50	1.33–1.95	0.188

IQR, interquartile ranges.

**Figure 3:**
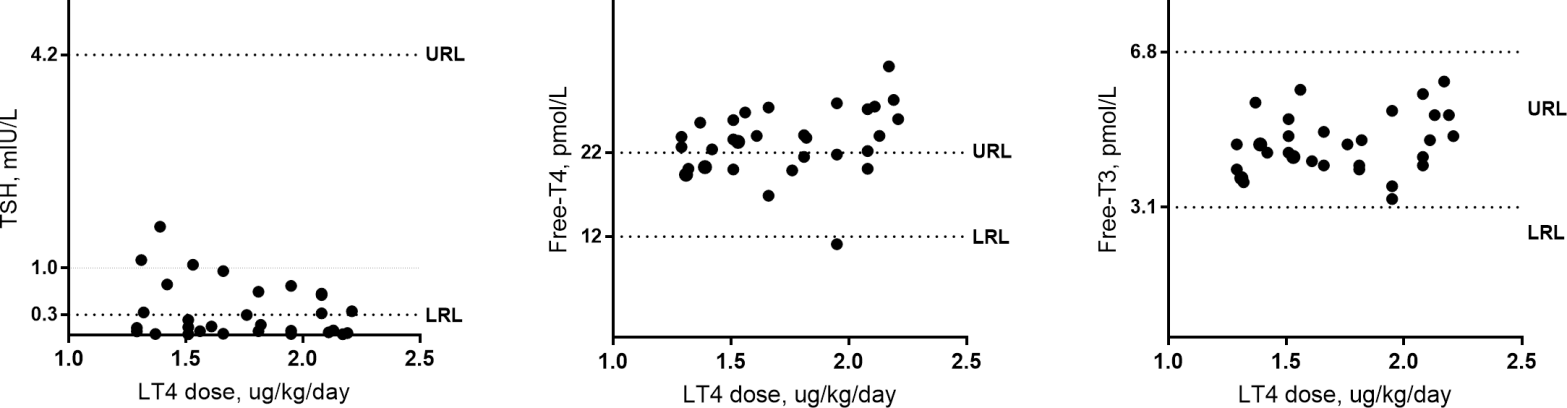
Correlation between LT4 requirements and thyroid hormone parameters.

**Figure 4:**
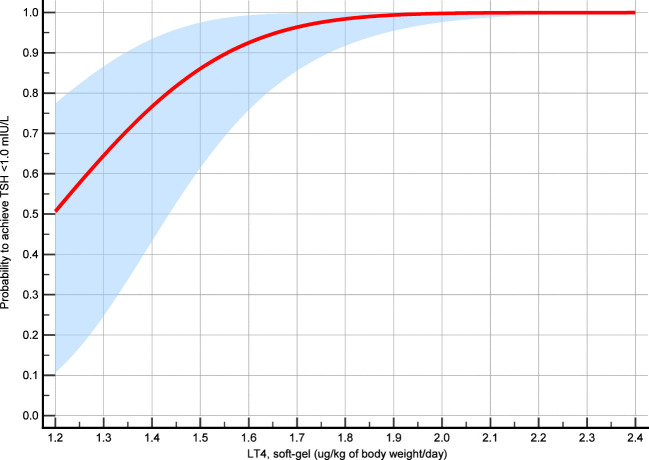
Probit regression model analysis. The probability of achieving TSH target was calculated according to the LT4 mean dose assumed during the study period and the mean TSH value recorded during that time interval.

Overall, the LT4 dose was significantly correlated with free-T4 (p = 0.03) but not with TSH (p = 0.42) and free-T3 (p = 0.13). **Figure 3** illustrates the correlation between LT4 levels and TSH, free-T3 and free-T4. Among the 30 patients, TSH was <1.0 mIU/L in 27 (90%) cases, <0.5 mIU/L in 21 (70%), and <0.1 mIU/L in 2 (6.7%). When the LT4 dose–TSH response effect is analyzed by probit regression model, a dose of 1.84 (95%CI 1.57–2.12) µg/kg/day showed 99% probability to achieve TSH target of <1.0 mIU/l. In addition, the probability to achieve the same TSH value was 75% with a dose of 1.38 µg/kg/day (95%CI 1.17–1.59) and 50% with a dose of 1.20 (95%CI 0.96–1.43). **Figure 4** shows the results of the probit regression model.

**Figure 5:**
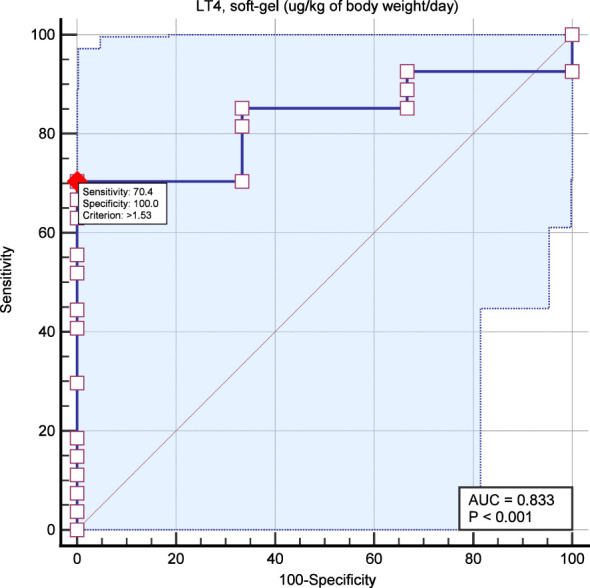
ROC curve analysis aimed to identify the most accurate LT4 dose for predicting TSH target (i.e., <1.0 mIU/L). The red dot indicates the most accurate point of LT4 dose to predict TSH <1.0 mIU/L (i.e., 1.53 ug/kg/day).

ROC curve analysis was performed considering the LT4 dose (µg/kg/day) and TSH <1.0 mIU/L. AUC was 0.83 with p <0.001. The most accurate cutoff of LT4 dose was 1.53 ug/kg/day with 70% sensitivity and 100% specificity (**Figure 5**).

## Discussion

Chronic hypothyroidism is a condition in which the risks and consequences are preventable with appropriate long-term treatment with LT4. As this drug has a narrow therapeutic index, individualization and titration of treatment are mandatory to achieve and maintain the therapeutic goal (25). The bioavailability of LT4 may depend on the characteristics of pharmaceutical preparations, such as excipient composition and stability over the shelf life (26), but also on the solubility properties that have been proven to be different among different preparations (4, 5). Most published studies comparing different LT4 formulations have focused on TSH changes obtained by switching from tablet to liquid or soft gel preparations. These studies demonstrated better TSH values following the switch and a reduced number of dose adjustments, resulting in higher patient satisfaction (13, 27) and better compliance with treatment and physicians, which is one of the major issues in long-term treatment (28, 29).

The present study was conceived to evaluate LT4-C performance in terms of achievement of target TSH at unchanged doses over time. Patients with athyreosis were chosen because of the virtual absence of a thyroid remnant. In addition, the TSH value of DTC patients is usually maintained within a strict range, regardless of whether it is suppressed. As a result, finding low-normal TSH levels in this DTC population is not rare. This setting was then reliable to evaluate the LT4-C dose-TSH response effect, with TSH <1.0 mIU/L as reference. The results showed that 1) all cases but two had TSH <1.0 mIU/L at a median LT4-C dose of 1.71 ug/kg/day, 2) the probability to achieve TSH <1.0 mIU/l was excellent (99%) with a dose of 1.84 ug/kg/day and very good (75%) with a dose of 1.38 ug/kg/day, and 3) the most reliable LT4-C dose threshold to achieve TSH <1.0 mIU/L was >1.53 ug/kg/day with good accuracy. As previously described, the median free-T4 value of our patients was slightly higher than the URL to maintain the appropriate free-T3 concentration in the absence of thyroid tissue and in patients with low-normal TSH levels (30). Furthermore, these values may be justified by the different hours of blood sampling that characterized our study group; unlike TSH values, free-T4 levels may be influenced by hormonal ingestion for the next 9 h (31). Further analysis per hour of therapy ingestion did not show any significant results (data not shown). The analysis of LT4-C requirement in elderly patients should confirm that the LT4 need is lower, even if it is not statistically significant due to the low number of patients and the slightly lower median value of median TSH in the elderly compared to younger patients (3, 21). Even though this is a retrospective study, the findings raise some questions about the recommendations of international guidelines for using LT4 (3). In general, hypothyroid patients with minimal endogenous thyroid function should require LT4 doses of 1.6 ug/kg–1.8 ug/kg of body weight and some studies estimated higher doses (i.e., 2.0 ug/kg–2.1 ug/kg). In addition, the guidelines discussed that DTC patients needing TSH suppression require a dose of 2.1 ug/kg–2.7 ug/kg (3, 21). Based on the present study, athyreotic DTC patients can start LT4-C therapy at a dose lower than that indicated in the guidelines. Furthermore, since our series did not show TSH levels higher than the upper reference limit, the probability of undertreatment was virtually zero.

We decided *a priori* to exclude patients taking drugs or having disorders potentially interfering with LT4 performance because many studies on these categories of patients have been carried out. One *in vitro* study showed better solubility of caps compared to different tablet preparations at increasing pH (5). These data were confirmed in patients with impaired gastric acidic output (15). Recently, the independence of the LT4-C requirement on gastric juice pH *in vivo* has been shown (9). In the present study, the better performance of LT4-C compared to the tablet preparation is evident, even after excluding patients with known features interfering with thyroxine treatment. A possible explanation for LT4-C better performance stems from possible variations in gastric acid production in the general population. Indeed, a paper published in 2010, evaluating gastric juice acidity in healthy subjects, reported that only 50% of them showed a gastric juice pH <2 (normal gastric juice pH) and 22% of these patients are characterized by actual hypoacidity (pH>3.5) (32). The notion that 80% of *H. pylori* infections, with a worldwide prevalence of 48%, go undetected despite all patients developing gastritis (33, 34), supports the usefulness of this formulation.

This study has some limitations. First, it was a retrospective study. This means that the enrolled patients did not receive a fixed predefined LT4 dose during their follow-up. Second, there was no comparator of the LT4 formulation (i.e., tablet, liquid, and generic). We then discuss only the results obtained with caps. Third, we used TSH <1.0 mIU/L as the reference to evaluate the LT4-C dose-TSH response effect. However, the included patients with DTC did not have a specific indication for this specific TSH target. The major strength of this study is that it included patients with athyreotic DTC, which represents a good model for evaluating the efficacy of LT4-C at a stable dose.

In conclusion, this study showed that patients with athyreotic DTC can be initially treated with an LT4-C dose lower than previously stated to achieve the appropriate target TSH in the majority of cases. Since the present data are retrospective and considering that the sample was small and included highly selected cases, these findings can be assumed to be reliable in athyreotic patients only. These data can help pave the way for further improving the challenge of individualized LT4 therapy, even if individual features such as age, sex, body weight, and metabolic status remain essential.

## Data availability statement

The raw data supporting the conclusions of this article will be made available by the authors, without undue reservation.

## Ethics statement

The study was approved by Tessin ethical committee. The studies were conducted in accordance with the local legislation and institutional requirements. The human samples used in this study were acquired from primarily isolated as part of your previous study for which ethical approval was obtained. Informed consent was obtained from all subjects involved in the study.

## Author contributions

PT: Conceptualization, Formal analysis, Methodology, Software, Validation, Visualization, Writing – original draft, Writing – review & editing. TP: Investigation, Methodology, Visualization, Writing – review & editing. ZD: Data curation, Writing – review & editing. IS: Data curation, Formal analysis, Visualization, Writing – review & editing. EG: Formal analysis, Validation, Writing – review & editing. LR: Data curation, Software, Writing – review & editing. CV: Supervision, Writing – review & editing.
